# Deciphering the molecular mechanism of water boiling at heterogeneous interfaces

**DOI:** 10.1038/s41598-021-99229-5

**Published:** 2021-10-06

**Authors:** Konstantinos Karalis, Dirk Zahn, Nikolaos I. Prasianakis, Bojan Niceno, Sergey V. Churakov

**Affiliations:** 1 Institute of Geological Sciences, University of Bern, 3012 Bern, Switzerland; 2 Lehrstuhl für Theoretische Chemie/Computer Chemie Centrum, Friedrich-Alexander Universität Erlangen-Nürnberg, Erlangen, Germany; 3 Laboratory for Waste Management (LES), Paul Scherrer Institute, 5232 Villigen, Switzerland; 4 Laboratory of Scientific Computing and Modelling (LSM), Paul Scherrer Institute, 5232 Villigen, Switzerland

**Keywords:** Molecular dynamics, Structure of solids and liquids

## Abstract

Water boiling control evolution of natural geothermal systems is widely exploited in industrial processes due to the unique non-linear thermophysical behavior. Even though the properties of water both in the liquid and gas state have been extensively studied experimentally and by numerical simulations, there is still a fundamental knowledge gap in understanding the mechanism of the heterogeneous nucleate boiling controlling evaporation and condensation. In this study, the molecular mechanism of bubble nucleation at the hydrophilic and hydrophobic solid–water interface was determined by performing unbiased molecular dynamics simulations using the transition path sampling scheme. Analyzing the liquid to vapor transition path, the initiation of small void cavities (vapor bubbles nuclei) and their subsequent merging mechanism, leading to successively growing vacuum domains (vapor phase), has been elucidated. The molecular mechanism and the boiling nucleation sites’ location are strongly dependent on the solid surface hydrophobicity and hydrophilicity. Then simulations reveal the impact of the surface functionality on the adsorbed thin water molecules film structuring and the location of high probability nucleation sites. Our findings provide molecular-scale insights into the computational aided design of new novel materials for more efficient heat removal and rationalizing the damage mechanisms.

## Introduction

Water, with its unique thermodynamic properties, which drastically change at the state points of phase transition^1^ is omnipresent in everyday life and natural processes^2^. Water is also one of the most widely used coolants and solvents in industrial applications^3,4^. Boiling is a first-order phase transition characterized by a discontinuity of the heat capacity. In high-temperature thermo-hydraulics, the cooling of material surfaces is particularly efficient in the boiling regime as long as the surface is covered with a film of liquid like water. At these conditions, the surface heat can be efficiently removed via the liquid phase and consumed through the evaporation process. At the critical heat flux (CHF), which is the limit of the safe operating conditions of a system^5^, the water film vaporizes (unrestrained bubble expansion parallel to the heated surface^5^) and the surface comes into contact with the vapor phase, causing the dry-out phenomenon^6,7^. The heat transfer rate is reduced and heat cannot be efficiently dissipated at such conditions, provoking a local temperature rise of the underlying surface material. The dry-out phenomenon has a devastating impact^5^ on the aging of the solid surface affecting the efficient heat removal (from solid to fluid), which potentially reduces the service life and the safe operation of industrial infrastructure^8,9^. Understanding the conditions under which cavities (seeds of the vapor bubbles) prefer to form and the presence of a thin adsorbed layer is crucial^10^ for the safe operation of power plants. Based on the fundamental understanding of the nucleation mechanism, new materials (both in terms of structure and functionality) can be developed with optimized functionality (i.e., efficient surface heat removal/exchange with the coolant). The boiling can be considered continuous evaporation upon the thermodynamic limit (above this limit, the liquid suffers instantaneous vaporization called explosive boiling^11^). At the initial stage of the low-density gas phase nucleation in the high-density liquid phase, the interface is substantial/distinct. The bubble nucleation and growth in the continuum scale framework can be described as mechanical work necessary to create an interface defined by the interface energy (surface tension) per unit of area. The definition of the interface, interface energy and surface area can be challenging at the molecular scale. The molecular-scale mechanism of the nucleation and condensation is not fully understood. Nucleation and stable growth of a bubble in homogeneous fluid require local density fluctuations and a cavity formation with critical bubble size. However, close to solid interfaces, the fluid properties radically change due to repulsive and attractive interactions based on the interface’s hydrophobic/hydrophilic functionality. These interactions are crucial for analyzing heterogeneous water nucleate boiling and cannot be addressed using traditional macroscopic techniques^12^.

The fundamental insight into the heterogeneous bubble nucleation mechanism is obtained by atomistic simulations. At the molecular level of interactions, evaporation (bubble nucleation) involves infrequent changes caused by intrinsic and infrequent fluctuations (a rare event). The absence of a free interface (vapor–liquid interface) requires crossing a nucleation barrier involving the emergence of sufficiently large vapor pockets within the metastable liquid^13^. The most promising path sampling techniques for analyzing rare events in atomic and molecular systems (in conjunction with molecular dynamics simulations) are transition path sampling (TPS)^14,15^, transition interface sampling (TIS)^16^, milestoning^17^, and forward flux sampling (FFS)^18^. The TPS scheme has been widely used to investigate a broad spectrum of dynamic processes (phase transitions^19–24^, reactions^25^ and conformational rearranges^26,27^). The TPS scheme uses a systematic approach for selecting dynamic trajectories that connect thermodynamic states of the system separated by a large activation barrier without introducing bias due to external driving forces, potential, or reaction coordinates^19,28^. The FFS scheme has been utilized for the analysis of superheated LJ fluids^29,30^ and evaporation in hydrophobic confinement^31–33^. The studies of heterogeneous nucleate boiling^34^ are limited to the method using such an external biasing field. More specifically, the nucleate boiling dynamics have been analyzed by creating artificial voids in the bulk^35^, introducing negative pressure^36^, superheat the fluid or the solid surface^37,38^ and trap gas in pits and scratches on the surface^39^. Molecular dynamics simulations have also utilized for the analysis of the explosive boiling^40–42^ and the onset boiling^43^. While the applied external bias helps to cross-reaction activation barrier, the effect of such artificial bias needs to be quantified^28^.

In this study, the heterogeneous water boiling mechanism on the monoclinic zirconia (used as thermal barrier coatings material, cladding material in the fuel assemblies of the nuclear power plants etc.) was investigated using the TPS molecular dynamics (MD) simulations. The main advantage of the TPS approach precut in this work is that the free energy surface of vapor nucleation is obtained based on self-consistent continuous atomic trajectory tracking the full-length dynamical pathways from liquid to vapor and vice versa. On this basis, an unprejudiced (without introducing bias and overheat) investigation of the dynamics of bubble nucleation and growth is enabled. The simulations provide insight into the effect of hydrophilic and hydrophobic interactions on the boiling mechanism, the formation of a thin molecular film of adsorbed water at fluid vapor interface and the nature of the vapor nucleation surface sites controlling.

## Results and discussion

**Figure 1:**
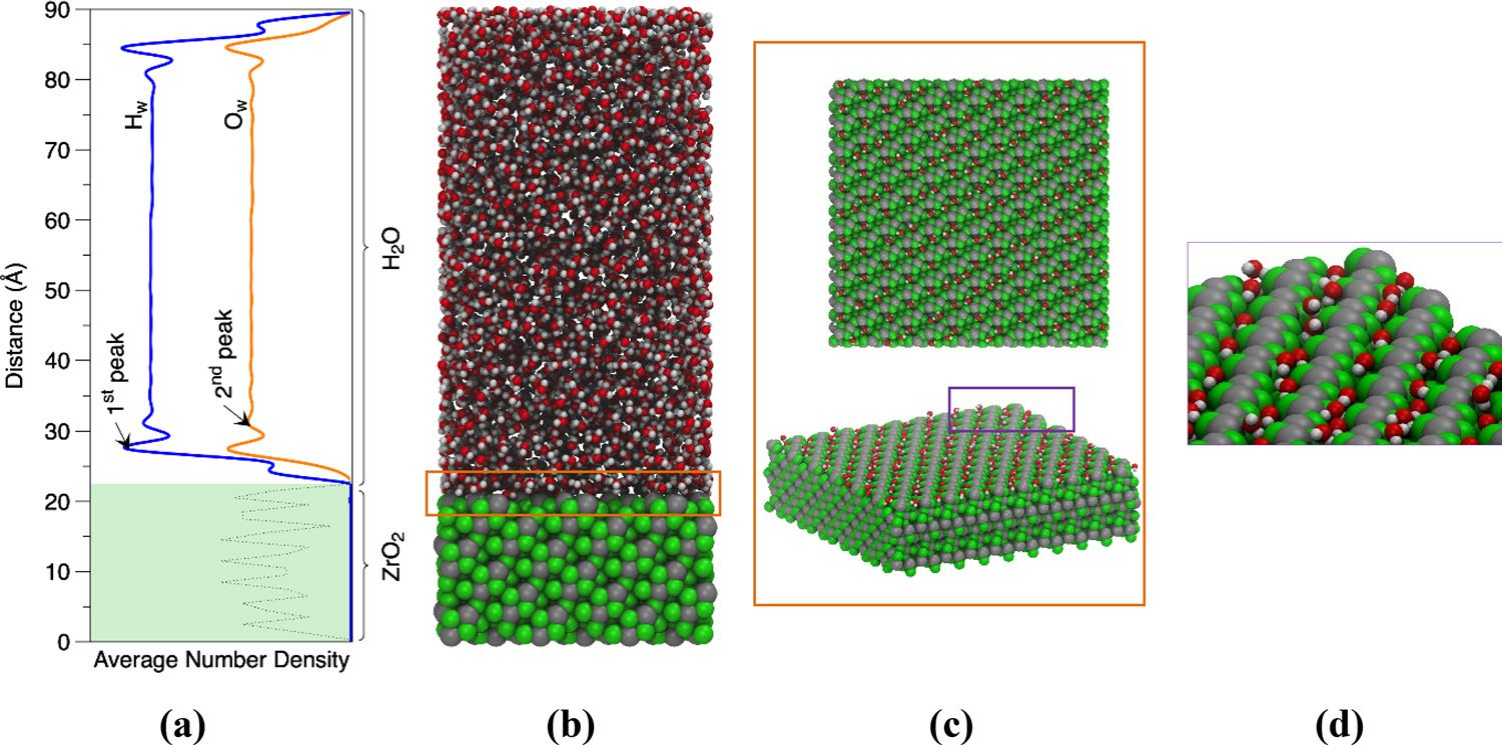
Illustrative representation of the system setup for the MD simulations of hydrophilic (− 111) surface of monoclinic baddeleyite (mZrO_2_) in equilibrium with water at 396.15 K. (**a**) density profile along the z-axis representing the parallel to zirconia interface solvent layers (**b**) the supercell used for the MD analysis of the heterogeneous water boiling, (**c**) top and perspective view of the (− 111) baddeleyite surface with only the first adsorbed layer of water molecules corresponding to the first oxygen maxima on the density profile and (**d**) magnified view portraying the orientation of the adsorbed water molecules. The oxygen atoms of the zirconia are green, the zirconium atoms are grey, the oxygen and hydrogen atoms of water are red white, respectively. Images were created using Visual Molecular Dynamics (VMD) v1.9.4^47^ software.

The surface functionality (hydrophobicity/hydrophilicity) can be analyzed based on the density profiles (number of hydration layers and the density variations, Fig. 1a) and the radial distribution function between specific water molecules and distinct surface functional groups (Fig. S1), showing the extent of attractive or repulsive interactions at the interface. The simulation supercell of the interface between water and (− 111) surface of monoclinic zirconia (mZrO_2_) is shown in Fig. 1b. The water structuring at the interface was examined at both ambient conditions (298 K) and at 396 K, corresponding to the boiling temperature of SPC/E water^44^. Water oxygen density profiles across the solid–fluid interface (see Fig. 1a) reveal two peaks at distances 3.1 Å and 6.2 Å from the outermost oxygen atoms’ position on the zirconia surface. In the water hydrogen density profile (Fig. 1a), a pre-peak located closer to the surface with an intensity similar to the oxygen intimates that one hydrogen bond per water molecule is formed between the H_2_O molecules and the solid interface. The density profile obtained for the (001) interface is remarkably different in respect to the (− 111) interface (Fig. S2), indicating, unlike surface functionality (hydrophobic). These highly structured (hydration layers) layers of water molecules^45^ are of crucial importance for the heat transfer phenomena (heat dissipation from the fluid). In the case of hydrophobic interfaces or very weak hydrophilic interfaces, the probability distribution’s intensity becomes negligible, potentially leading to the formation of voids (high nucleation density^46^) adjacent to the interface, interrupting the heat flux and likely causing the surface to overheat. The fluid structuring of the (− 111) interface results from an interplay between oxygen and surface zirconium atoms’ arrangement forming the diagonal channel occupied by the water molecules (Fig. 1c). The positively and negatively charged atoms at the zirconia surface create a heterogeneous electric field, which affects the dipole orientation of the water molecules. Between the zirconium atoms at (− 111) surface are the primary adsorption surface sites (see Fig. 1c) responsible for the first layer of the adsorbed thin water film. The water molecules in the first adsorbed layer are oriented with their one positive charge (dangling hydrogen atom) closer to the solid interface (Fig. 1d), advocating that the attractive coulombic interactions from the zirconia oxygen atoms dominate. Based on the Radial Distribution Functions, RDFs (Fig. S1) it was computed that the distance between oxygen atoms of the water molecules (${O}_{w}$) and oxygen and zirconium atoms of the zirconia (${O}_{Zr{O}_{2}}$ and Zr, respectively) are 2.90 Å and 2.97 Å, respectively. Comparably, the distance between the hydrogen atoms of the water molecules and the ${O}_{Zr{O}_{2}}$ and Zr was calculated to 1.72 Å and 3.53 Å, respectively. The average coordination number between ${O}_{w}{-}{O}_{w}$ in the adsorbed thin water film is smaller (under coordinated) in comparison to the bulk water and equal to 1.14. The coordination number between the ${O}_{Zr}{-}{H}_{w}$ which obtained from the integral of the first peak in the RDFs were determined equal to 3.2 and 0.9 for the (− 111) and (001) interfaces, respectively. These values indicate the number of hydrogen atoms that are on the ${O}_{Zr}$ solvation sphere.

**Figure 2:**
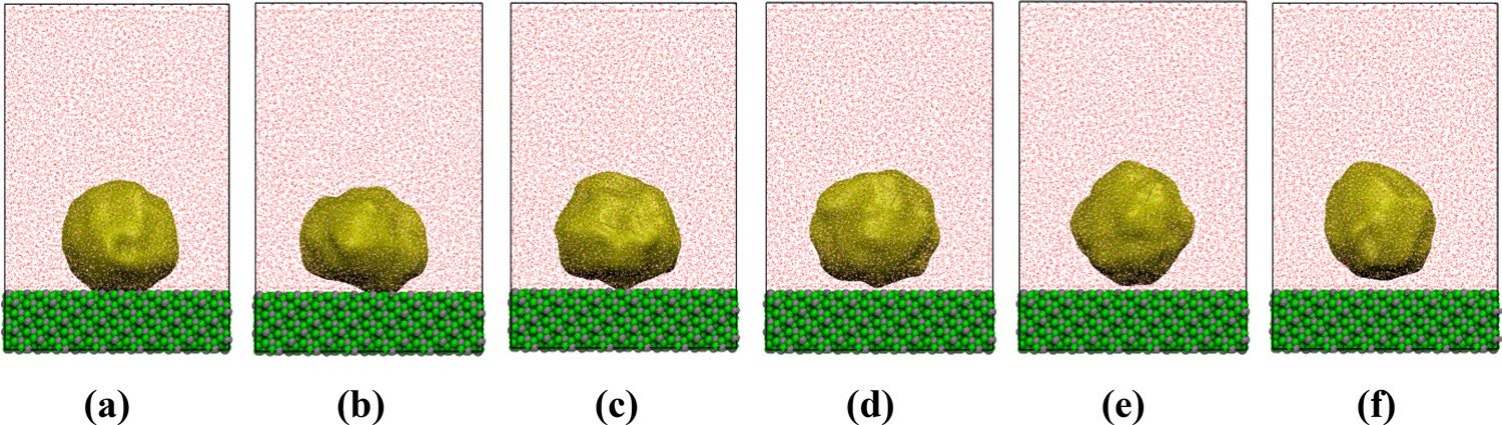
Time evolution of a vapor bubble on the zirconia (− 111) interface (frames **a**–**f**). The vapor bubble was found to maintain the spherical shape across the trajectory. In (**b–d**) frames, a meniscus was created as expected in the macroscopic conceptualization of vapor bubble (**b**) and finally, the bubble detached from the interface (frames (**e,f**)). In the latter frames, a non-evaporating thin water film was adsorbed on the zirconia interface (see also (**b**)) due to the surface hydrophilicity. The snapshots correspond to simulation times of 0.05, 0.10, 0.15, 0.30, 0.35 and 0.40 ns respectively. Images were created using VMD v1.9.4^47^.

**Figure 3:**
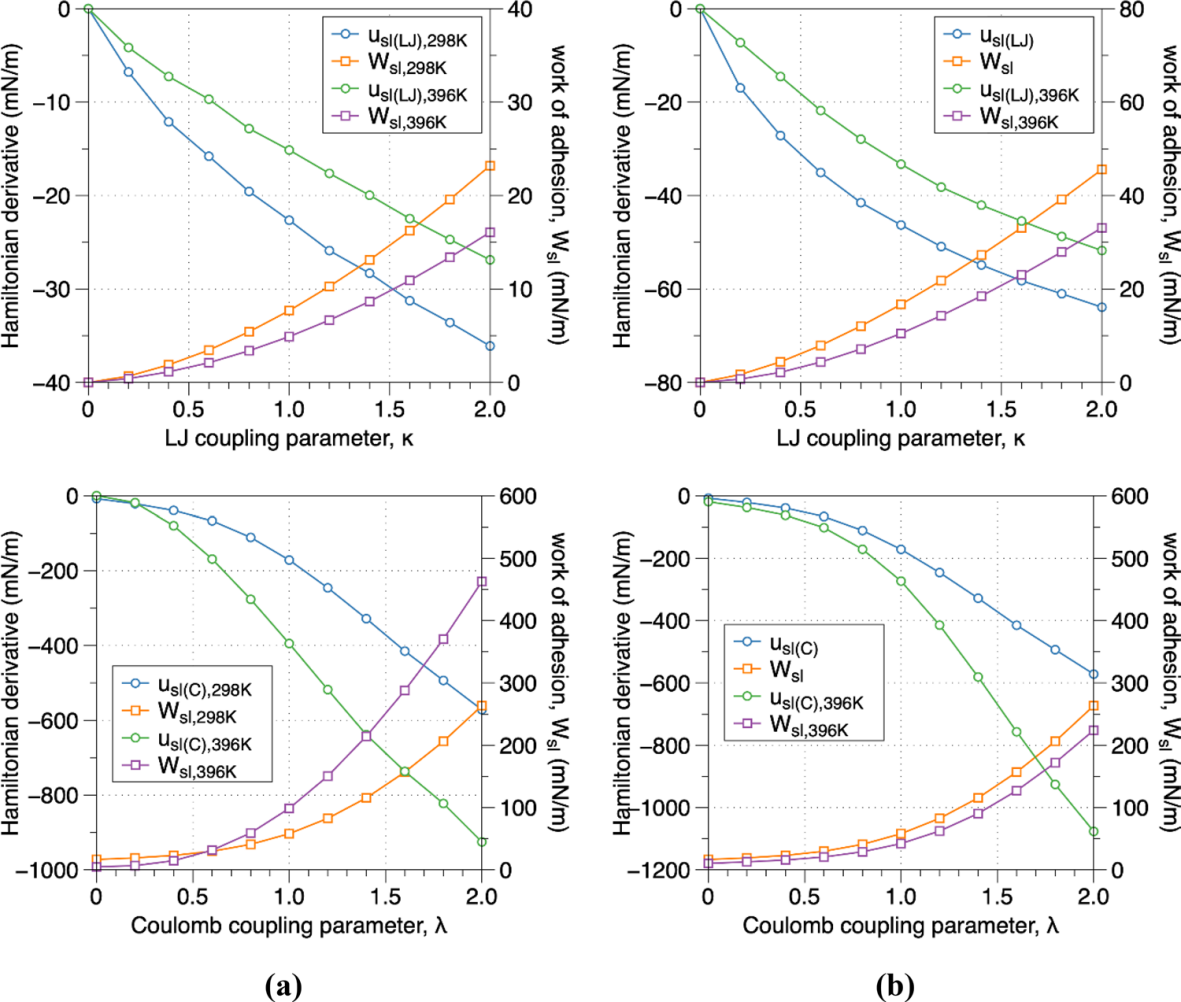
Average of Hamiltonian derivatives (u_sl(LJ_) and u_sl(C)_)and work of adhesion (W_sl_) for (**a**) the zirconia (− 111) interface plane at 298.15 K and 396.15 K and (**b**) the zirconia (001) interface plane at 298.15 K and 396.15 K respectively. The work of adhesion (W_sl_) at κ = 1 on the LJ coupling parameter case was used as the initial value for the work of adhesion using the Coulomb coupling parameter. The cumulative W_sl_ (at λ equal to 1.0) of the Coulomb coupling parameter was applied in the Young-Dupré equation to calculate the water contact angle.

The surface tension and the functionality (hydrophobicity/philicity) of (− 111) and (001) surfaces were determined by analyzing the contact angle of a vapor bubble embedded in a condensed phase at the interface^46^. The conceptualization of the boiling interface applied in the macroscopic simulations is demonstrated in Fig. S3^48^. It is based on the existence of a hydrophilic surface at close to boiling temperature conditions (< 10 K of the boiling temperature) that a macroscopic bubble is separated from the solid surface by a thin water film (~ 1 mm thick), except for a very narrow interval in the middle of the bubble. The thickness of the film depends on the specific hydrodynamic conditions, water diffusivity and heat flux^10,49^. Based on the aforementioned representation, one can distinguish the apparent contact angle and the microlayer with the evaporating and non-evaporating thin-film regions. The atomistic simulation applied in this work cover the nanometer scale phenomena at the solid/fluid interface, addressing the intrinsic “nano” meniscus formed on the interface (Fig. S3)^4,48,50^. For the contact angle analysis, two different methodologies were utilized; in the first approach (Fig. 2, Suppl Figs. S4, S5), an artificial vacuum bubble was generated on the interface by subtracting water molecules and in the second approach the method developed by Surblys et al.^51^ was applied (Fig. 3). Using the first approach (artificially generated vacuum domain—bubble), the average contact angle (Fig. 2a–e) of water in contact with the (− 111) plane at ambient conditions and before a thin water layer adsorbed on the interface (Fig. 2f) was calculated equal to 139.2 ± 7°. The initial vacuum domain (bubble) due to the surface tension, transformed from the initial cubic shape to an intermediate irregular shape and finally to a spherical shape. The hydrophilic functionality of the (− 111) interface, lead to the adsorption of water molecules on the solid interface forming a bubble meniscus (Fig. 2b–d) which progressively eliminated, leading to the formation of a thin-water film (Fig. 2e,f). The height of the thin water film is approximately 7 Å which suggests that the bubble preferential site is located on the PMF minimum (Fig. S7). Using the method developed by Surblys and based on the Young-Dupré equation (${W}_{sl}={\gamma }_{lv}\left(1+cos\theta \right)$, where ${W}_{sl}$ is the work of adhesion, ${\gamma }_{lv}$ is the liquid–vapor surface tension and $\theta$ is the contact angle) the contact angles of (001) and (− 111) interfaces at 298.15 K and 396.15 K were obtained. Based on the results, it was determined that the (001) interface plane is hydrophobic (contact angle equal to 95° at ambient conditions). In comparison, the (− 111) interface plane is hydrophilic (contact angle equal to 145.5° and 167.0° for temperatures 298.15 K and 396.15 K, respectively). The hydrophilicity of the most favorable interface plane of mZrO_2_ (− 111) was also verified with the theoretical prediction based on the interatomic potential (Lennard–Jones) energy parameters which were used (ε_wall-liquid_/liquid > 1)^39^.

**Figure 4:**
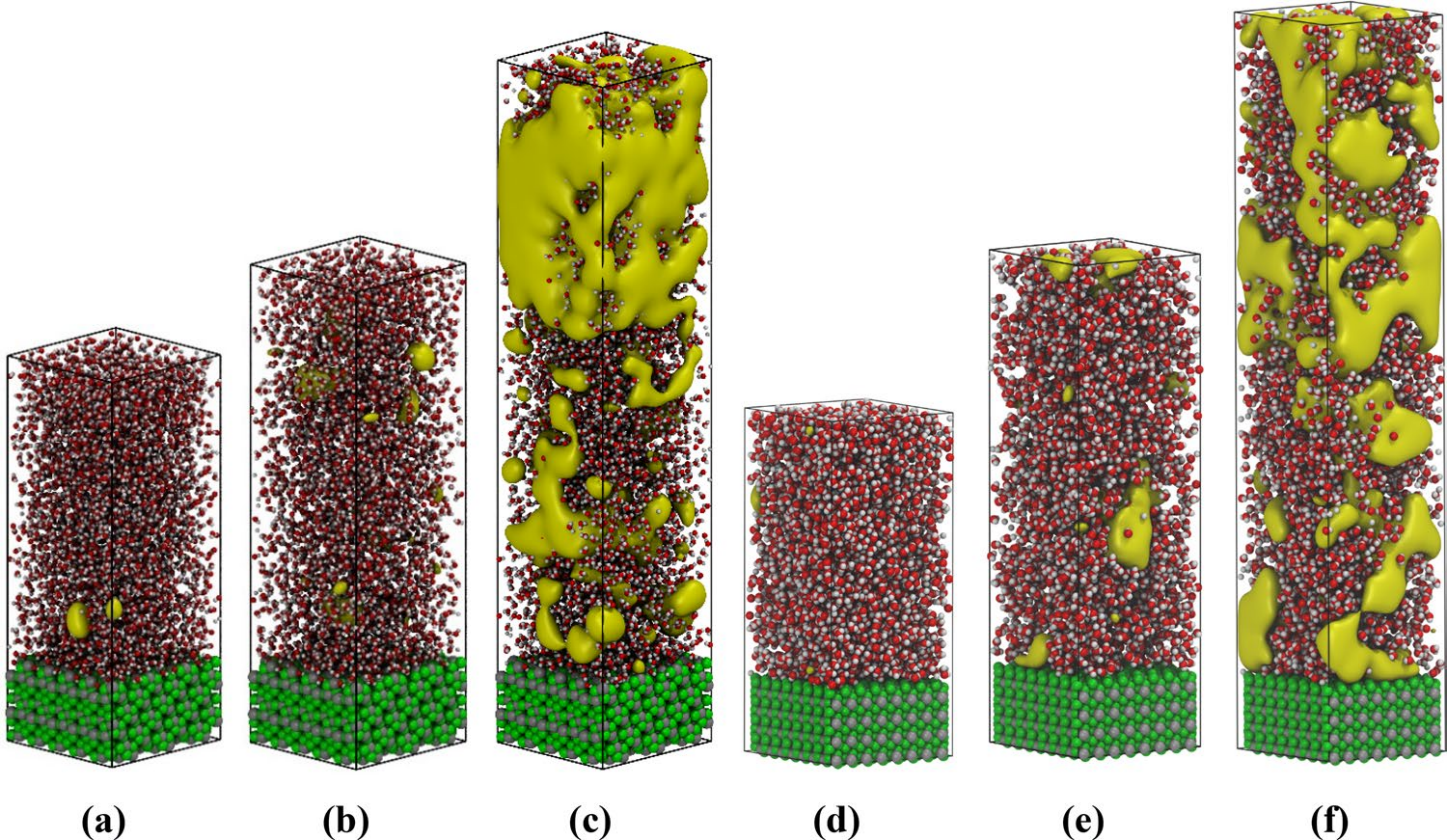
Explosive boiling configurations were obtained by overheating the system from 396 to 800 K using two different mZrO_2_ surface planes. The yellow iso-surfaces represent the voids inside the water phase. Voids are defined as a set of points in space separated by at least 3 Å distance from any water molecule^19^. The subfigures (**a–c**) correspond to the interface (− 111) and the (**d–f**) to the (001). It was observed that in the simulations with the (001) surface, a larger number of cavities are formed at the solid liquid-interface indicating the hydrophobic character of this interface. See Fig. 1 capture for the description of the color scheme. Images were created using VMD v1.9.4^47^.

Bubble nucleation is a rare event associated with a large activation energy barrier. Spontaneous formation of the vapor cavities at the boiling temperature cannot be observed in conventional MD simulations due to the rarity of such fluctuations and the wait time between consecutive events^13^. Consequently, the Transition Path Sampling (TPS) approach (see the TPS subsections) was applied to understand the bubble nucleation mechanism in the liquid–vapor transition at the vapor–liquid-solid interface. The TPS approach’s initial step is to generate a first, non-equilibrium trajectory leading from the liquid into the gas phase. The latter is achieved by overheating the equilibrated system from 396 to 800 K with a constant rate of 1 K/ps^19^. During the heating, the water density decreases progressively and at a certain point of a few picosecond duration the fluid density drops from 0.6 to 0.1 g/cm^3^. This sharp density drop is characterized as explosive (massive) boiling in which complex-shaped randomly distributed cavities (bubbles) emerge in the simulation domain (see Fig. 4).

In the explosive boiling trajectories (Fig. 4), the transition from liquid to gas (evaporation) is swift (in the range of some picoseconds), the kinetic energy of the molecules is not describing the boiling temperature and consequently, the bubble nucleation does not represent the boiling mechanism at the thermodynamic conditions of vapor–liquid–solid equilibria. These configurations’ most common characteristics (corresponding to the explosive boiling), merge randomly generated cavities into single, insulating water monomers and clusters.

**Figure 5:**
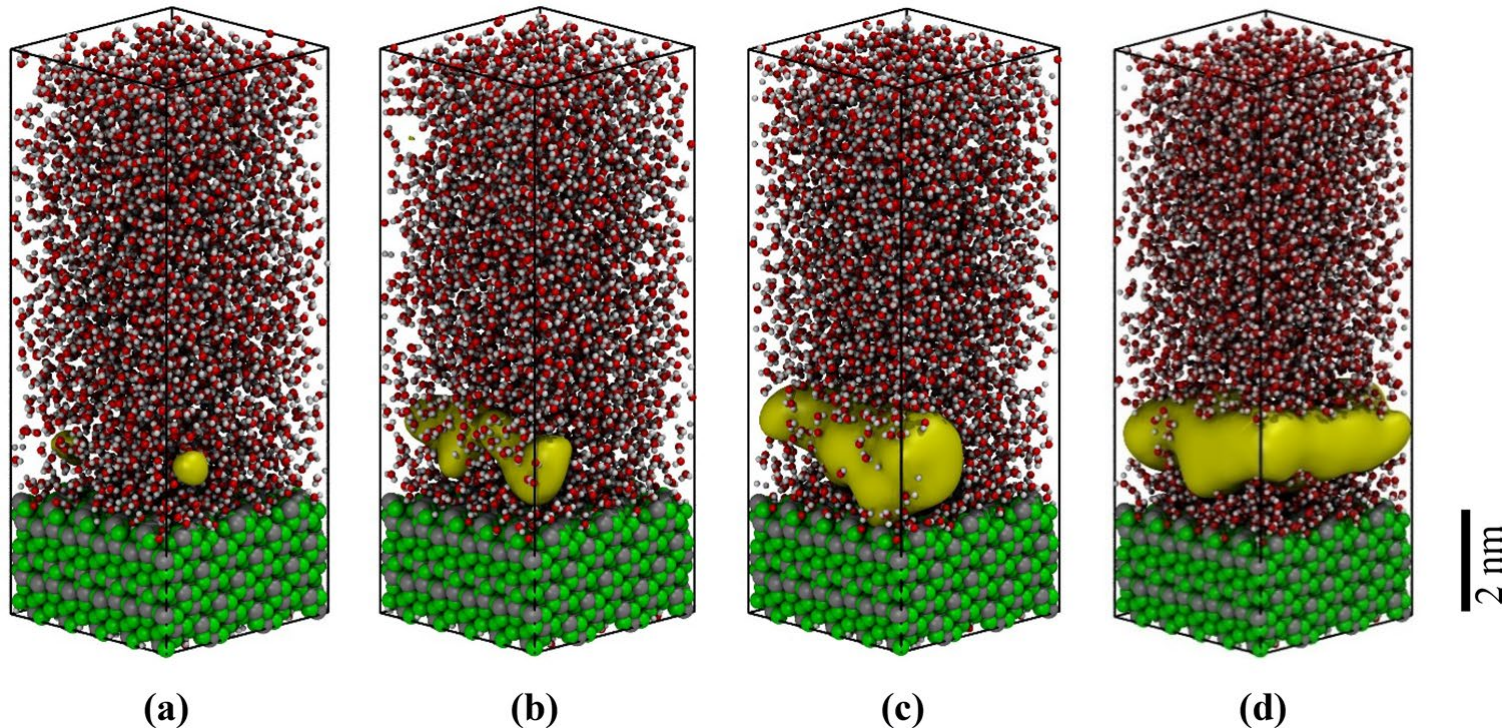
Representation of the initial stage of heterogeneous water nucleate boiling in contact with the hydrophilic (− 111) interface of mZrO_2_. An initial void at distance ~ 7 A from the solid interface nucleates (**a**) which grows (**b,c**) until the formation of a void layer (**d**). See Fig. 1 and 4 capture for further details. Images were created using VMD v1.9.4^47^.

**Figure 6:**
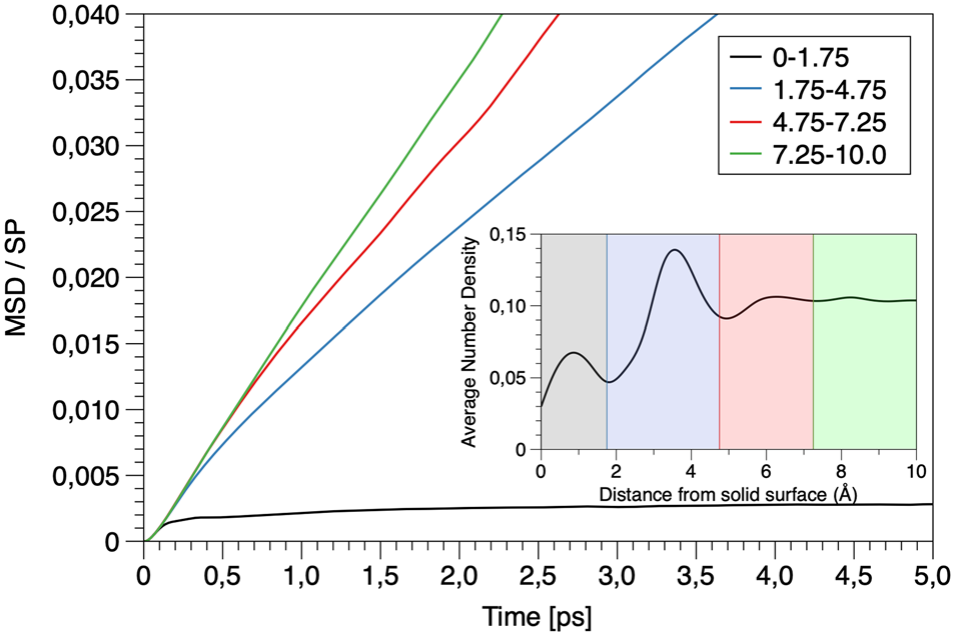
Parallel mean square displacement (MSD) divided by the survival probability (SP) for specific water layers from the zirconia interface at (− 111) face. The parallel MSD is defined as the average MSD values in x and y directions. The self-diffusion coefficients for the four layers are 8.06 × 10^−13^ m^2^/s, 1.6 × 10^−9^ m^2^/s, 2.4 × 10^−9^ m^2^/s and 4.02 × 10^−9^ m^2^/s, respectively.

Explosive boiling trajectories do not represent the equilibrium mechanism of vapor–liquid transition at the boiling temperature. These trajectories are just used to initiate the equilibration of the system and not considered for the sampling of the boiling mechanism. From the artificially biased generated trajectories describing the water evaporation (see Fig. 4), several trajectories were selected and propagated forward and backward in time with random velocity modifications (shooting, see subsection TPS in “Methods” section). A sufficiently wide range of shooting points and iterations was used to achieve the transition path trajectories’ decorrelation (Fig. S6). Based on the decorrelated trajectories, the dynamical pathway of boiling and condensation can be determined in comparably larger timescales in the range of some nanoseconds. At this time scale, the transition from densities of approximately 0.9 g/cm^3^ (in which there are no cavities) to the densities close to 0.3 g/cm^3^ (correspond to the vapor density) was efficiently sampled. Figure 5 shows a representative set of snapshots illustrating the vapor nucleation’s consequent steps and boiling at a constant temperature. Based on these configurations, the heterogeneous water boiling mechanism at liquid–vapor transition temperature and the nucleation location was analyzed. Cavities (diameter higher than 3 Å) are absent at liquid-like densities of 0.9 g/cm^3^ (Fig. 5a, apart from the solid surface). After several hundreds of picoseconds, multiple small cavities are formed in bulk. The formed cavities break the hydrogen-bonded network of the bulk water and act as a trigger for the formation of more cavities, which progressively merge and create a large vacuum domain (Fig. 5d). Analysis of the nucleation center distribution shows that the highly hydrophilic solid interface is covered with a thin film of water molecules. The cavities do not nucleate at the interface between the solid and liquid phase but between the ordered film of water at the surface and bulk like water located ~ 7 Å away from the solid surface (see the time evolution of density profile distributions in Fig. S6). The analysis of the water self-diffusion parallel to the surface^52–55^ (Fig. 6) revealed that the water molecules in the solid interface’s vicinity have much lower mobility than bulk-like water. The strong surface water interaction leads to the high structuring of water dipoles. These water molecules at the interface have a surface-controlled structural arrangement incongruent to the structure of bulk water. Consequently, the formation of bubble nuclei is anticipated at the weakly bound interface between highly ordered and bulk-like water layers (Fig. 5b–d).

In the first stages of the boiling process, subcritical vapor bubbles (cavities with a radius smaller than 5 Å) are formed in the liquid domain and subsequently disappeared (condensate). At steady-state conditions, all bubbles with a radius smaller than 10 Å are expected to collapse since the Young–Laplace pressure is smaller than the total pressure of the system^35,56^. Consequently, the TPS approach allowed us to visualize tiny radius cavities’ nucleation for the first time suggesting that the transition from the liquid to the vapor phase is triggered by nucleation and subsequent growth of the vapor phase seeds^23^. As observed in the configurations of Fig. 5, a liquid film of an approximate width of 7 Å is separating the vapor phase from the solid surface. In the course of the simulation, instant vacuum domains emerge in the vicinity of the solid interface (Fig. 5c). The lifetime of these vacuum domains was limited (some femtoseconds) due to the adsorption (condensation) of water molecules on the interface, regenerating the thin liquid film. To reveal the distinct thermodynamic parameters controlling the bubble nucleation at the hydrophobic and hydrophilic interfaces, the potential of mean force (PMF) acting on a hydrophobic LJ particle was calculated (Fig. S7). The LJ particle with an effective radius of 3.166 Å represents a vapor nucleus located at different distances to the interface. At the hydrophobic (001) interface, the PMF has a global minimum at the solid/fluid interface. This observation is consistent with the assumption that the water layer’s structure on the hydrophobic surface is similar to the water structure at the vapor–liquid interface. Therefore, the least amount is required for the formation of the water–vapor interface at the solid–liquid interface. On the contrary, at the hydrophilic (− 111) interface, the PMF has local and global minima at distances ~ 4.4 Å and ~ 7.1 Å, respectively. Accordingly, the thermodynamically most favorable location for the bubble nucleation is in the interface between structured water film at the surface and the bulk-like water domain. Indeed, the cavities were observed to nucleate close to the surface and extend at distances more extensive than the global minima of the PMF. The final bubbles were expanding and oscillating at the equilibrium distance of 13 Å from the interface, having a meniscus shape along the sampled trajectories. Based on the coordination number analysis, the coordination number of the surrounding water molecules of a bubble is not changed in the initial nucleation steps. Similarly, based on each water molecule’s kinetic energy, it was determined that the local temperature of the molecules surrounding the bubbles and those in the liquid bulk domain is oscillating around the same value (equal to the boiling temperature). The surrounding water molecules in the cavities are oriented with the one hydrogen atom in the normal direction to the cavity^57^. Also, the hydrogen bonds in the vicinity of the bubble are rearranged^57^.

In several applications, the most critical phenomenon controlling the heat exchange between the solid surface and the fluid is a thin molecular film of adsorbed liquid at the interface. The presence of such film is critical for the efficient removal of surface heat via evaporation. If vacuum domains are formed on the solid interface, the heat flux from the solid to the fluid is interrupted, leading to local solid overheat. The latter accelerates solid aging (uncontrolled oxidation), which affects heat removal efficiency via evaporation, the solid material lifetime, and, most importantly, the operation’s safety. Consequently, based on the current study’s fundamental knowledge, MD simulations using the TPS approach provide useful insights regarding the most appropriate solid materials (structural and functional properties), which will have the optimum characteristics for more efficient water boiling.

In conclusion, the heterogeneous water nucleate boiling in respect to the surface functionality was analyzed by utilizing the TPS approach using the MD method. Based on the state-of-the-art TPS approach, we were able to elucidate for the first time the heterogeneous formation and initial nucleation of phase of the vapor bubbles at the interface. The surface functionality was determined by calculating the vapor bubble contact angle on the solid interface. It could be shown that the nucleation of the cavities at the hydrophilic (− 111) plane takes is energetically more favorable at the interface of the structured water film adsorbed in the interface and bulk like water. This region corresponds to the minimum of the potential energy for the cavity nucleation for the hydrophilic interface. Accordingly, the hydrophilic surface remains covered by approximately two water layers, which favors the heat conduction and prevents the surface dry-out phenomenon on the surface. Contrary, the vapor nucleation at the hydrophobic (001) plane occurs at the interface between the solid and the liquid. This region corresponds to the minimum of the potential energy for the cavity nucleation for the hydrophobic interface. The contact angle understanding on different surfaces at nano-scales, which have been conducted in this work, gives boundary conditions for the film dynamics on the micro-scale, which in turn affects the apparent contact angle of the bubble and consequently the wetting of the surface. In turn, wetting has a significant impact on the departure from nucleate boiling and transition to film boiling, essential for many technological devices’ safe operation. Based on the obtained results, boiling models on scales larger than nano can be improved and more effective materials can be designed. The molecular-scale insight into the mechanism on the heterogeneous boiling phenomena obtained in this work opens up opportunities for the innovative design of boiling resilient, functional surfaces.

## Methods

### Interatomic potentials

MD simulations of heterogeneous water boiling have been performed using the non-polarizable SPC/E^58^ water model and the interaction potential developed by Martins et al. for the monoclinic zirconia (baddeleyite)^45^. The SPC/E model offers a compromise between a high level of accuracy and computational efficiency for large-scale simulations. More specifically, in respect to bulk properties, SPC/E water model reasonably predicts all the physical properties (density, compressibility, self-diffusion coefficient and dielectric constant). The Lorentz-Berthelot combining rule was used to determine the unlike Lennard–Jones parameters^59–61^.

### Molecular dynamics simulations

Molecular Dynamics simulations of heterogeneous water boiling were performed using LAMMPS v.2019 code^62^. Finite-size effects were examined by analyzing two different orthogonal supercells with considerably different dimensions consisting of a total of 11.664 and 84.672 atoms, respectively. In the first setup, the supercell was composed of 2168 H_2_O molecules and 720 ZrO_2_ formula units, while in the second setup, it was composed of 25.344 H_2_O molecules and 2.880 ZrO_2_ formula units. Periodic boundary conditions in all dimensions were applied. The system was equilibrated in the NP_z_T ensemble with approximate initial box dimensions of 38 × 38 × 90 Å and 75 × 75 × 160 Å, respectively. The geometry of the SPC-water held fixed using the rigid body approach^20,63^. An integration time step of 0.5 fs was used to ensure energy conservation and time-reversibility^61^, which is considered essential for the path-sampling scheme^21,22^. The temperature was controlled using a Nosé-Hoover thermostat with a relaxation time of 2.5 ps. The pressure was controlled using an isotropic Parrinello-Rahman barostat with a relaxation time of 10 ps to ensure that the liquid–vapor transition is not affected by volume fluctuations (avoiding condensation phenomena)^19^. The damped shifted force model with a 16 Å cut-off was used for the electrostatic interactions^64^. The applied temperature was 396 K, corresponding to the boiling temperature SPC/E water model^44^ as obtained from the Clausius–Clapeyron equation.

### Transition path sampling (TPS)

Transition path sampling (TPS) simulations^14,15,65^ were performed using an in-house code interfacing with LAMMPS. The initial trajectory connecting liquid and vapor state was obtained from an explosive boiling event by heating the water molecules at a rate of 1 K/ps from 300 to 800 K (line 1 in Fig. S8). From this trajectory, several snapshots were collected and propagated in both directions (forward and backward in time, lines 2–4 in Fig. S8, in order to reach the gas and liquid state, respectively) by applying the SPC/E water boiling temperature of 396 K^44^. If the selected configuration managed to relax to either state, the new trajectories were then generated iteratively by selecting a configuration from the preceding trajectory and slightly modifying the atomic momenta (shooting)^22^. The modified configuration, after ensuring that it conserves the linear and angular momentum and the total kinetic energy (velocities rescaling^25^) was then propagated in both directions of time. Changes in the atomic positions were not applied and consequently, the potential energy was conserved. This iterative approach was performed multiple times, ensuring the trajectory decorrelation from the artificially generated initial trajectory (Fig. S6). The total simulation time for each shooting was 1 ns. The water density and the average number of hydrogen bonds were used to classify the water molecules’ state.

### Potential of mean force

The potential of mean force (PMF) provides a measure of the difference in free energy between two states as a function of one or several degrees of freedom (i.e. distance between two particles)^66^. The PMF calculated in Fig. S7 indicate the potential energy difference for a hydrophobic particle representing a void as function of the distance to the interface. The minimum of the PFM represents the distance at which the void formation is energetically most favorable. The equilibrium PMF was calculated by a constraint force method on a hydrophobic particle. The hydrophobic particle with the water molecules’ LJ parameters was positioned with constrained z-distances from the solid-surface in steps of 0.1 Å and allowed to equilibrate on each slab for 0.5 ns. In the x–y plane, the particle was allowed to move in order to capture the potential global minimum of the specific slab. The PMF was obtained by integrating the mean force acting on the hydrophobic particle along the force-distance (z-distance from the solid surface) curve.

### Materials

As solid material, we selected the zirconium alloy, which is used in several applications (i.e. as cladding material of fuel rods in nuclear reactors). The zirconium alloy at normal conditions oxidized and becomes covered with a passivating layer of monoclinic mineral baddeleyite (mZrO_2_). Zirconia at low temperatures (< 1400 K) has monoclinic phase^45,67^. In the MD simulations, the zirconium alloy surface was represented by the most stable facet (− 111)^68,69^. The (− 111) plane was while the (001) plane was hydrophobic. The surface structure (different orientation of the atomic planes and surface roughness) affects the hydrophobicity/hydrophilicity of the interface and consequently affects the bubble nucleation mechanism^70,71^. The hydrophilic surfaces provide favorable conditions for bubble nucleation and formation of vapor films^70^.

## Supplementary Information

Supplementary Information.

## Data Availability

Our codes for the contact angle calculation and the input files for the TPS simulations are available from the corresponding authors upon request.
