# Supplementary material for: Deciphering the molecular mechanism of water boiling at heterogeneous interfaces
Source: Sci Rep. 2021 Oct 6;11:19858. doi: 10.1038/s41598-021-99229-5 (PMC8494797; doi:10.1038/s41598-021-99229-5)
Supplement: Supplementary file 1 — Supplementary Information. [file 41598_2021_99229_MOESM1_ESM.pdf]

Deciphering the molecular mechanism of water boiling at heterogeneous interfaces

Konstantinos Karalis[1]*, Dirk Zahn[2], N. Prasianakis[3], B. Niceno[4] and Sergey V. Churakov[1,3]*

[1]Institute of Geological Sciences, University of Bern, CH-3012 Bern, Switzerland
[2]Lehrstuhl für Theoretische Chemie/Computer Chemie Centrum, Friedrich-Alexander Universität Erlangen-Nürnberg, Erlangen, Germany
[3]Laboratory for Waste Management (LES), Paul Scherrer Institute, CH-5232 Villigen, Switzerland
[4]Laboratory of Scientific Computing and Modelling (LSM), Paul Scherrer Institute, CH-5232 Villigen, Switzerland
*Corresponding author(s): konstantinos.karalis@geo.unibe.ch, sergey.churakov@psi.ch

Supplementary Information

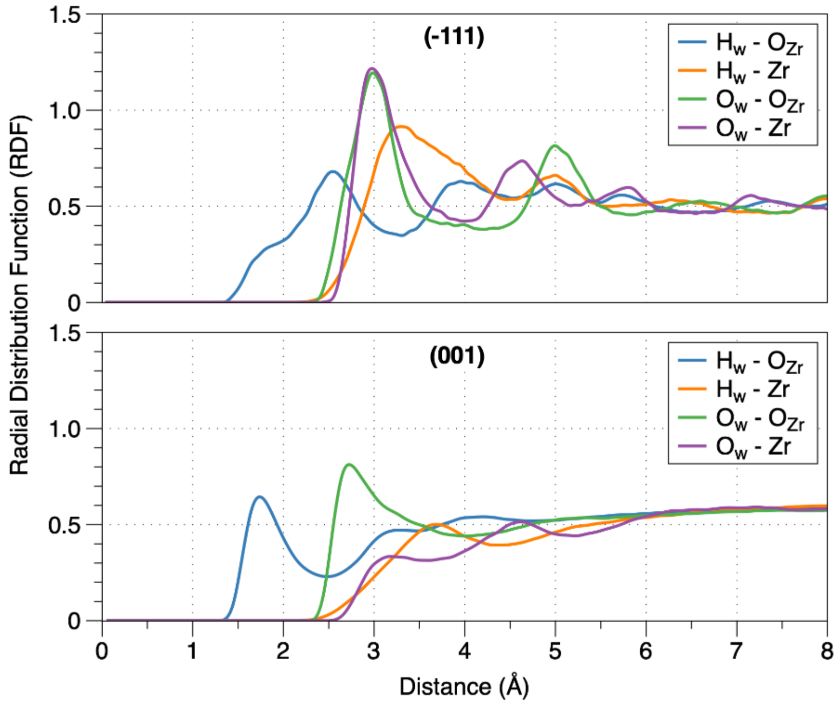

Figure S1. Radial distribution function of the zirconia interface atoms (Zr, O_{zr}) with the oxygen and hydrogen atoms of the water molecules for the (001) and (-111) interface planes. In both interface planes, the water dipoles are oriented with the hydrogen atom being closer to the interface.

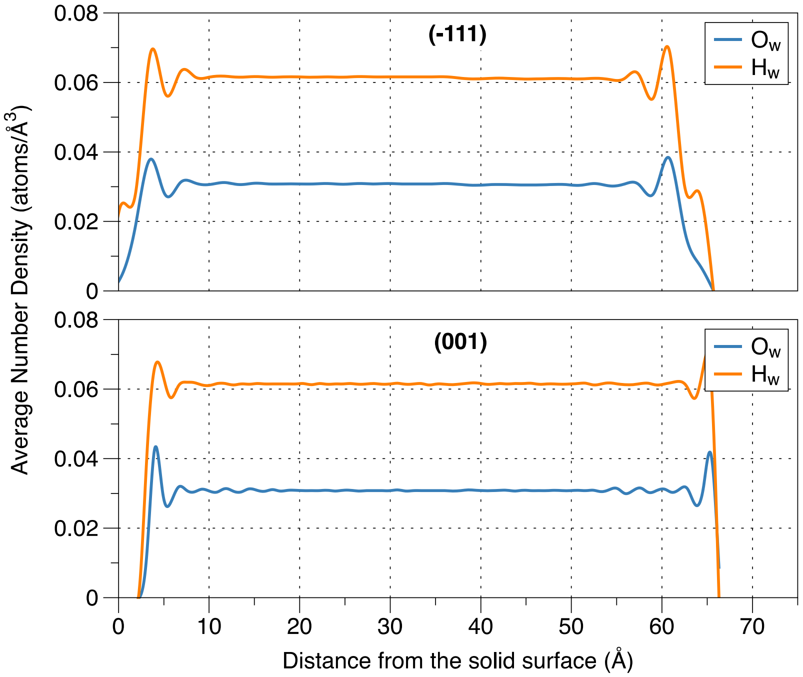

Figure S2. Density profile in fluid phase normal to zirconia interface at hydrophobic (001) and hydrophilic (-111) zirconia surface faces. In the hydrophilic (-111) interface plane, a hydrogen pre-peak close to the solid interface is evident, indicating the formation of hydrogen bonds between the water and solid atoms.

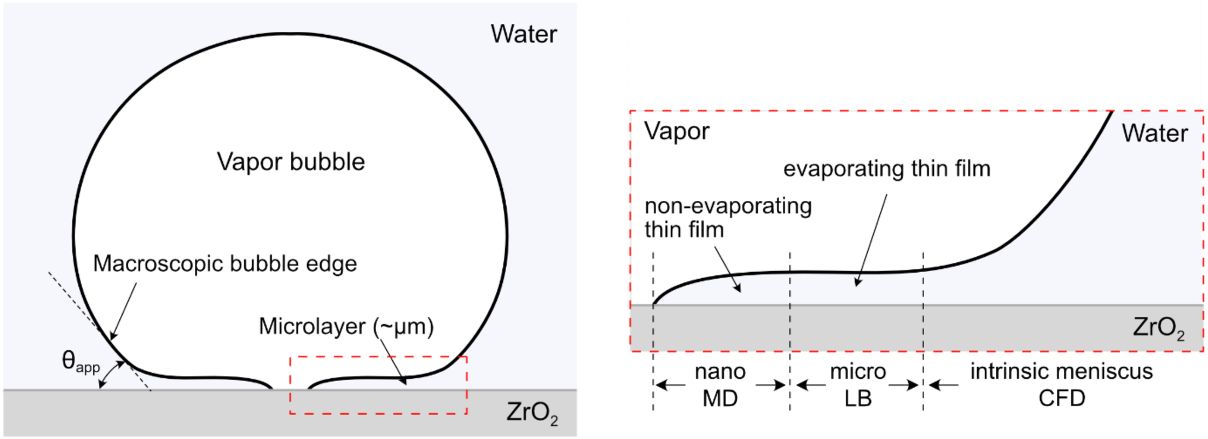

(a) **(b)**

Figure S3. a) Conceptual representation of a macroscopic vapor bubble on the zirconia interface and b) detailed presentation of surface vapor interface[4,43,45]. Molecular simulations (MD) capture the nucleation and evolution contact angle in wetting film in the nano-region. The micro-region and the formation of the intrinsic meniscus can be capture by performing simulations in the mesoscale (Lattice-Boltzmann, LB and Computational Fluid Dynamics, CFD).

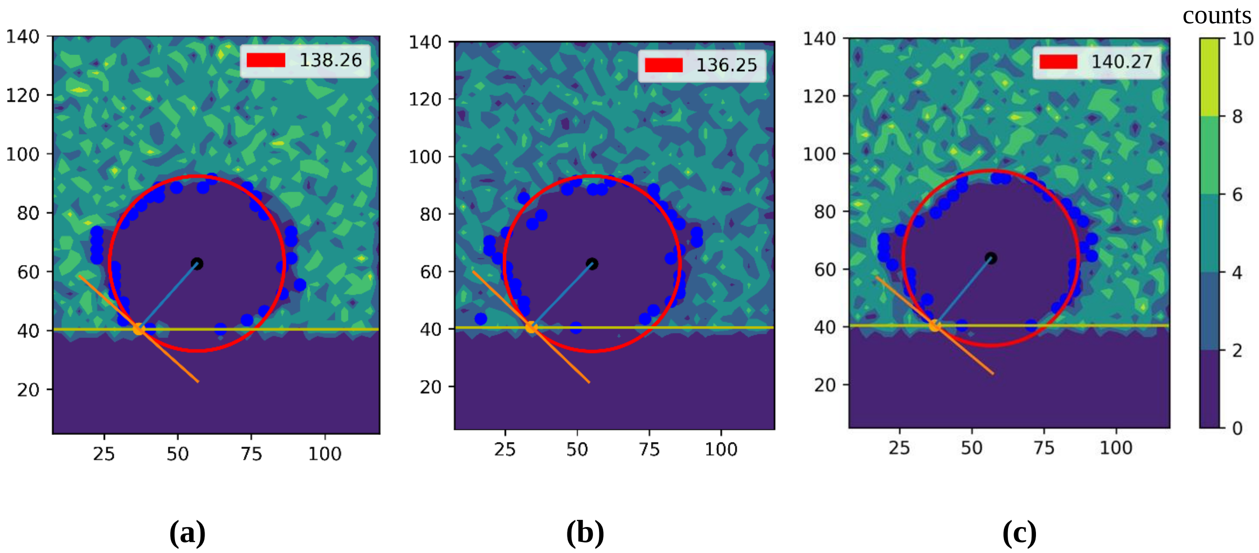

Figure S4. Contact angle estimation on water vapor bubble embedded into condensed phase at (-111) the interface for three different timesteps (a)-(c). The blue points define the liquid/void interface and used for the fitting procedure.

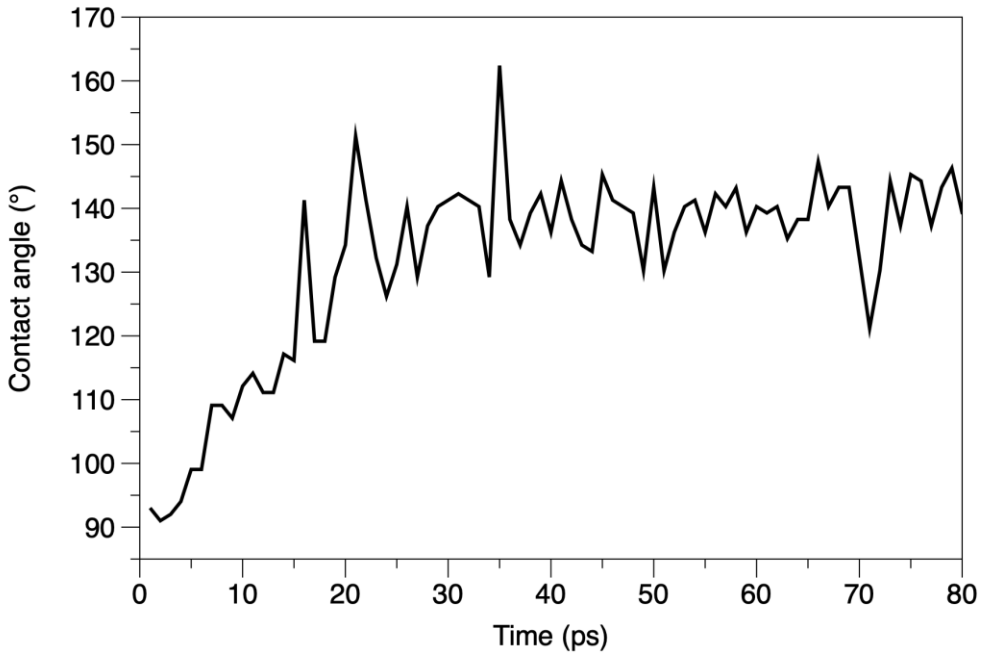

Figure S5. Contact angle variation of vapor bubble in respect to time.

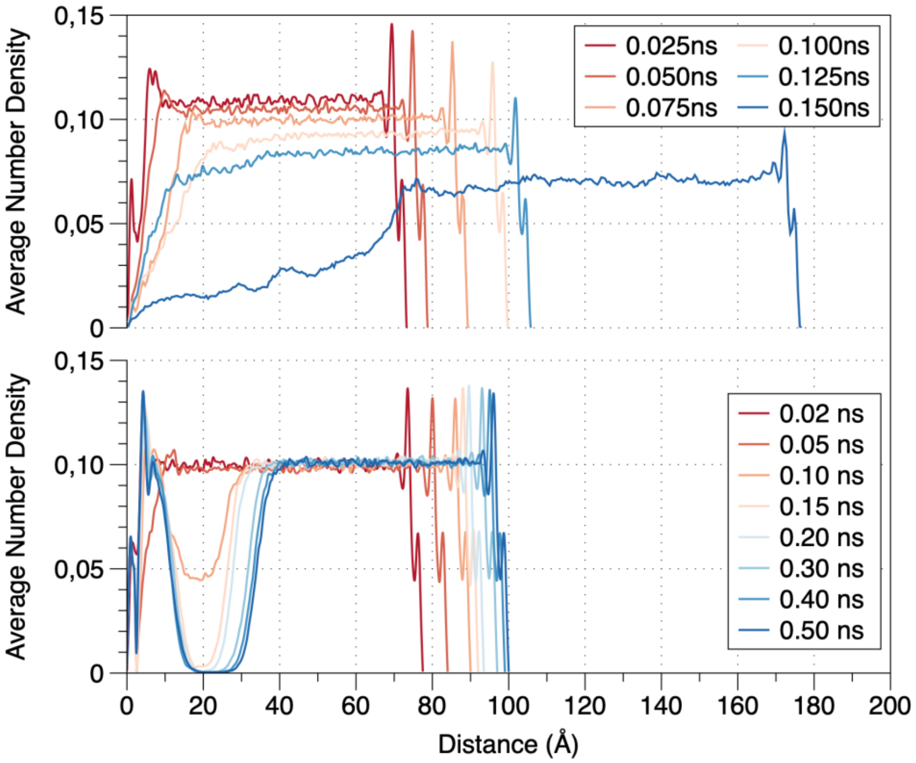

Figure S6. Water density profile evolution during the initial stage heterogeneous water nucleate boiling in contact with the (-111) interface of $mZrO_2$. The two independent density profiles indicate a thin adsorbed water layer (6-8Å) on the hydrophilic interface. The almost constant increase of the box size along z-distance (bottom graph) indicates the trajectory decorrelation from the artificially generated pathway (the initial stage of heterogeneous water nucleate boiling, explosive boiling).

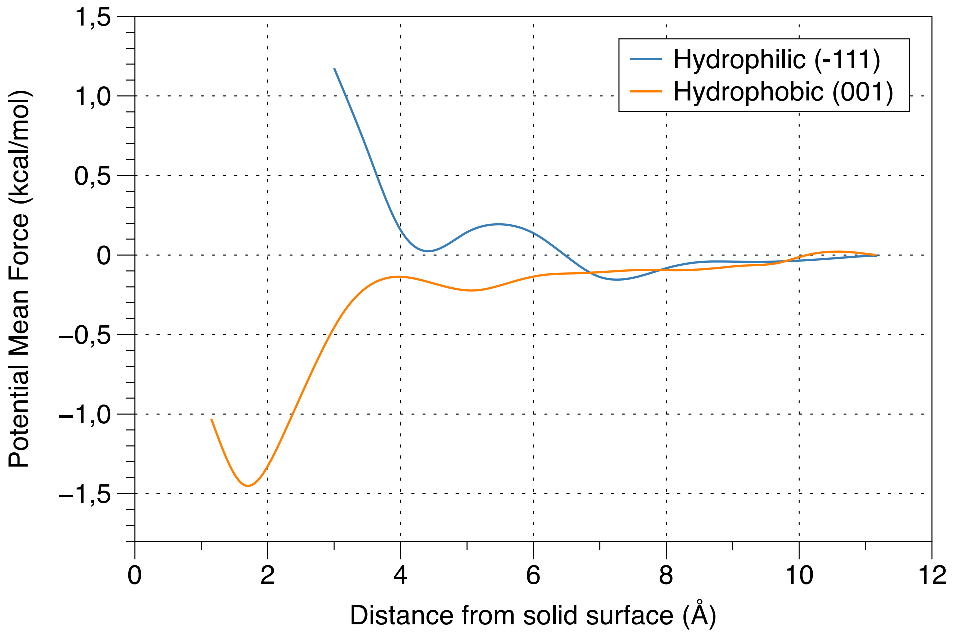

Figure S7. Potential of mean force (PMF) profile of a hydrophobic particle normal to zirconia interface at (001) and (-111) surface faces.

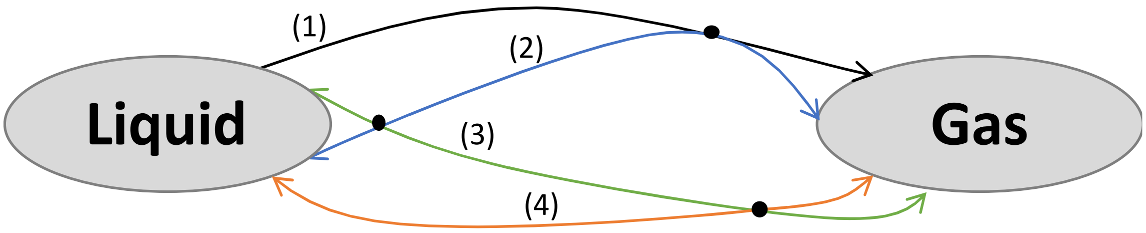

Figure S8. Schematic representation of the transition path sampling (TPS) shooting approach between two stable states (liquid and gas). The shooting approach is used to create new paths (i.e. lines 2-4) in which the momenta are perturbed and the equations of motion are integrated backward and forward in time.
